# Conceptualization and Inventory of the Sexual and Psychological Burden of Women With Pelvic Floor Complaints; A Mixed-Method Study

**DOI:** 10.1016/j.esxm.2022.100504

**Published:** 2022-03-23

**Authors:** Alma M. Brand, Scott Rosas, Wim Waterink, Slavi Stoyanov, Jacques J.D.M. van Lankveld

**Affiliations:** 1 Faculty of Psychology, Open University of The Netherlands, Heerlen, The Netherlands; 2 Faculty of Educational Sciences, Open University of The Netherlands, Heerlen, The Netherlands; 3 Concept Systems, Inc., Ithaca, NY, USA

**Keywords:** Pelvic Floor Complaints, Restrictions, Distress, Group Concept Mapping, Sexual and Psychological Burden

## Abstract

**Introduction:**

Despite the fact that the literature reports various restrictions and types of distress in women with pelvic floor complaints, a comprehensive overview of women's sexual and psychological burden emerging from these complaints is lacking, which compromises our ability to assess and grasp the impact to women.

**Aim:**

This study was performed to conceptualize women's sexual and psychological burden and create a more comprehensive overview on this topic from both women's and health care providers’ perspectives. Furthermore, this research intended to identify items to populate a to-be-developed instrument to assess sexual and psychological burden.

**Methods:**

In Group Concept Mapping, 125 statements were used about restrictions and distress that women with pelvic floor complaints experienced. Women with, and health care providers with and without pelvic floor complaints (13 women and 3 men) sorted the statements into comprehensive self-labeled clusters and rated their nature and severity. Multidimensional scaling and hierarchical cluster analyses were performed to identify a conceptual model of coherent clusters of statements. Item-total correlations of severity scores were calculated to identify statements that can be used in future research to represent women's sexual and psychological burden.

**Main Outcome Measure:**

A conceptual model emerged, and outcomes of item-total correlations were then examined again using the conceptual model.

**Results:**

Seven distress clusters were identified, namely, loss of control, sexual distress, feeling insecure, feeling wronged, feeling helpless, feeling angry, and feeling disappointed. Feeling insecure appeared more pervasive than other distresses. Furthermore, 33 statements were identified that can be used in future research to develop an instrument to assess sexual and psychological burden representing both women's and health care providers’ perspectives.

**Conclusion:**

The conceptual model and list of statements may concisely represent the sexual and psychological burden of women with pelvic floor complaints from both women's and health care providers’ perspectives on this topic.

**Brand AM, Rosas S, Waterink W, et al. Conceptualization and Inventory of the Sexual and Psychological Burden of Women With Pelvic Floor Complaints; A Mixed-Method Study. Sex Med 2022;10:100504.**

## INTRODUCTION

Women with pelvic floor complaints experience different types of restrictions and distress in their daily, social, and sexual functioning, and in their intimate relationships.^1^, ^2^, ^3^, ^4^, ^5^, ^6^, ^7^, ^8^, ^9^ Complaints, such as incontinence, pelvic organ prolapse, and pelvic and genital pain complaints are common. These complaints can affect sexual functioning by provoking painful intercourse, and problems involving sexual desire, arousal, and orgasm which often cause sexual distress.^10^^,^^11^ Pelvic floor complaints often require lifestyle changes and adjustments for which some women seek help, while others do not.^3^^,^^6^^,^^8^^,^^12^, ^13^, ^14^, ^15^, ^16^ A comprehensive overview of women's sexual and psychological burden with pelvic floor complaints is currently lacking, compromising our ability to inventory and understand the impact on women.

Women often feel insecure because of their loss of control over pelvic floor muscle function and the resulting complaints.^17^ Incontinence often leads to embarrassment, fear of others noticing the accidents, frustration, social isolation, and a decrease in self-confidence, challenging daily, social and sexual activities.^8^^,^^16^^,^^18^ Furthermore, pelvic organ prolapse often involves diminished body image and alterations in self-perception.^8^^,^^19^^,^^20^ Women who experience this type of distress tend to report a low genital self-image and to feel sexually unattractive.^8^^,^^21^, ^22^, ^23^ In addition, pelvic and genital pain involves loss of sexual pleasure and challenges women's intimate relationships.^24^, ^25^, ^26^ Indeed, higher distress levels are associated with lower relationship quality.^27^

Besides insecurity, genital and pelvic pain can cause anxiety and depression,^17^,^22^,^28^, ^29^, ^30^, ^31^, ^32^, ^33^, ^34^ and the women concerned also express shame and guilt over their accompanying sexual problems.^25^^,^^35^ Furthermore, painful intercourse often reduces sexual interest, affecting both sexual pleasure and sexual satisfaction.^9^^,^^35^^,^^36^ Specifically, pregnancy- and childbirth-related pelvic floor complaints appear to involve challenges, restrictions, and distress related to the specific physical condition of the women concerned.^37^ After giving birth, some women with pelvic floor complaints have difficulty coping with pain, and feel insecure about their future.^38^ All these separate findings reflect a fragmented overview of the restrictions and distress that women with pelvic floor complaints experience.

A previous qualitative analysis by our research group of restrictions and distress in daily, social and sexual functioning, and in intimate relationships of pregnant, parous, and nulliparous women with pelvic floor complaints text-mining analyses of interviews has provided a more comprehensive overview that highlights sexual problems as an important topic of discussion.^39^ Additionally, it seems important to have an instrument available that accurately and validly assesses the sexual and psychological burden of women with pelvic floor complaints. Therefore, a concise and comprehensive overview of the multidimensional content of women's restrictions and distress with pelvic floor complaints is needed. In this study, the results of previous research on this topic are condensed and sorted to develop a conceptual model of women's sexual and psychological burden with pelvic floor complaints. To develop a clear and understandable instrument for women with pelvic floor complaints and their health care providers, both their perspectives on this topic need to be included. The emergent conceptual model will provide a firm base for a new instrument that can be further developed and validated in future research.

At this stage of knowledge building in this field a qualitative method that retains the complexity of the studied phenomena as much as possible, including women's personal experiences, is preferable over a quantitative approach that inevitably entails a reduction of the richnss and complexity of the data. Therefore, the present study builds from interview data of previous mixed-method research.^39^ To accomplish the binary goal, the use of another mixed-method study design called Group Concept Mapping (GCM) was chosen. This structured method was used to identify the relationship between large quantities of concepts and ideas, to create conceptual models from qualitative data to provide insight into how a group views a topic or construct of interest.^40^, ^41^, ^42^, ^43^, ^44^ Therefore, this method seems most appropriate to pursue the aims of this study. The first aim is to develop a structured conceptual model that represents women's sexual and psychological burden with pelvic floor complaints including both women's and health care providers’ perspectives. The second aim is to use this model to derive a concise list of statements that can be used in future research aimed to develop an instrument to assess the sexual and psychological burden of women with pelvic floor complaints.

## MATERIAL AND METHODS

### Study Design

The online platform groupwisdom^45^ is a web application for GCM analyses and is used to integrate the qualitative group processes with multivariate statistical analyses to help express and define concepts and their interrelationships.^40^^,^^41^ Typically, GCM consists of a number of successive steps, namely idea generation such as brainstorming, the synthesis of ideas, the sorting and rating of statements, multivariate analyses, and the interpretation of the output.^43^^,^^46^

As a post hoc analysis of the emergent conceptual model, a selection process was undertaken to identify statements that can be used in future research for inventory development.^42^^,^^44^ By following the procedure for item selection in measure development outlined by Nunnally,^47^ a core set of statements from the conceptual model were selected. The selected statements were examined again against this conceptual model to check their distribution.

### Participants and Recruitment

Twenty persons were invited to participate in the GCM-procedure. To gain input from different perspectives, at least 7 women with current or past experience with pelvic floor complaints were included. Six women were recruited from a pelvic physical therapy practice, and 3 participated in the interviews that provided input for this study. Four of these women were also health care provider. Because subgroup comparisons were not planned due to the limited sample size, we did not question the other participants about personal experience with pelvic floor complaints. These health care providers, including 3 male professionals possessed a diverse range of expertise in women's health. 17 invitees agreed to participate. Eventually, a total of 16 participants, including at least 6 women with personal experience with pelvic floor complaints sorted and rated statements. Three of these women were also health care providers.

## DATA PREPARATION AND COLLECTION

### Idea Generation

Although GCM typically employs brainstorming as a technique for idea generation, other means to generate input for analysis are acceptable, such as interviews or document reviews.^43^ For this study, input for sorting and rating was extracted from interviews of women with pelvic floor complaints that were conducted in a previous study of our research group.^39^ However, the input, in the form of single word concepts that were needed in the previous analyses,^39^ proved unsuitable for inventory development, because of their lack of context, and - consequently - the risk of misinterpretation. Therefore, building from the interview data of the previous study,^39^ in this study, concepts were put back into their specific context in the form of short statements to make sure all relevant issues were included before further analysis. From the initial 866 statements that consisted of reported restrictions and distress with pelvic floor complaints duplicates were removed, and similar or overlapping statements were merged. The remaining 242 statements were independently categorized by 3 members of the research team into 6 categories representing, respectively, physical, daily, social, sexual, relational, and psychological issues to gain a general idea about salient topics per category.

The research team reached immediate consensus about the categorization of 122 out of these 242 statements. Based on majority voting, another 109 out of the 242 statements were categorized. Two statements were deleted and the remaining 9 were categorized subsequent to discussion and consensus. The list of 240 statements contained, respectively, 20 physical, 25 daily, 27 social, 46 sexual, 13 relational, and 109 psychological statements per category. Similar statements in each category were merged in another round to obtain a diverse 125-item statement list. The final unstructured statements were uploaded to the project website in groupwisdom,^45^ where the statements were randomized for each participant, before each sorting and rating task.

### Sorting and Rating

The experts were then asked to sort statements into groups based on their ideas about their similarity to one another. The sorting task is unstructured, meaning that participants can arrange the content in any number of groups they consider reasonable and logical.^43^ The sorting task was framed using several guidelines, namely, there could not be 1 group including all statements, nor could each statement be put in its own group, although some statements could end up as a single-item group. Furthermore, statements could only be placed in 1 group, and it was not allowed to make a group of statements that did not belong anywhere else. Participants were asked to label each group in terms that best described the included statements. On completion, the experts rated the statements as restriction or distress, and the severity of each statement. Restrictions vs distress ratings involved a binary choice, in which “1” was used to indicate a restriction, and “2” to indicate distress. Severity ratings were scored on a 5-point Likert-type scale ranging from 1, indicating “not severe,” to 5, indicating “very severe.”

### Data Collection Procedures

The Ethical Review Board of the Open University of the Netherlands granted ethical clearance for the study protocol in May 2019. The participants were sent an email with the link to the project website, including an information letter and the informed consent form. Participants accepted the informed consent form online, and further instructions about the sorting and rating tasks were also provided online. The tasks were estimated to take approximately 2 hours. The data collection procedure was tested before the start of the process to check the clarity of the instructions. Data collection took place over a 6-week period in November and December 2020.

## DATA ANALYSES

### Sorting

GCM utilizes multiple quantitative data analyses of the sorted data, specifically multidimensional scaling (MDS) and hierarchical cluster analysis (HCA).^43^^,^^46^^,^^48^ The MDS analysis produces a configuration of the statements on 2 dimensions (x,y). This configuration is based on the principle that frequently combined statements are most often located near each other, whereas those combined less frequently are more distant. A total similarity matrix is compiled and represents how often each statement is sorted onto the same pile by the participants, which automatically implies that the highest score on any pile cannot exceed the total number of participants. The total similarity matrix is analyzed using MDS, and the convergence of the data was judged using the final stress value. In the process of convergence, the clearest configuration is sought, based on as little iterations as possible, to obtain a clear 2-dimensional visualization of the position of each statement in relation to other statements. The stress value indicates the fit of this configuration with the original similarity matrix. Lower stress values reflect a stronger convergence between the optimal and actual 2-dimensional configurations.^46^^,^^49^

HCA of the MDS-coordinates, applying Ward's algorithm,^43^ provided the basis for cluster definition, and cluster map creation. There were no specific selection criteria for the final number of clusters, therefore, a successive higher-to-lower review strategy was used following suggestions by Kane and Trochim.^41^ Thus, to refine the range of choices, 15 through 4 cluster solutions were evaluated separately by the research team to define which cluster configuration appeared to most adequately represent the participants’ ideas and suggestions.

### Rating

The rating analyses in this study were used to provide insight into the participants’ perspectives on the classification of statements as restriction or distress, and the severity of the statements. The average cluster rating scores were generated by first averaging the rating values across participants, and then by averaging the mean scores of the statements within each cluster. A relative pattern match graph was produced to visualize how each cluster corresponded in nature and severity in comparison with other clusters, based on the minimum and maximum average scores.

Furthermore, the severity rating scores were used in the item selection process for an inventory to assess the psychological burden of physical complaints. These ratings were entered into SPSS-26 for the computation of item-total correlation statistics. Typically, in item selection procedures, Nunnally recommends excluding items with an item-total correlation below the threshold of 0.40.^47^ However, in this study, statements were initially included with an item-total correlation of 0.70 or higher, due to the relatively high mean severity scores, and the large number of statements.^42^

## RESULTS

### The Conceptual Model

**Figure 1:**
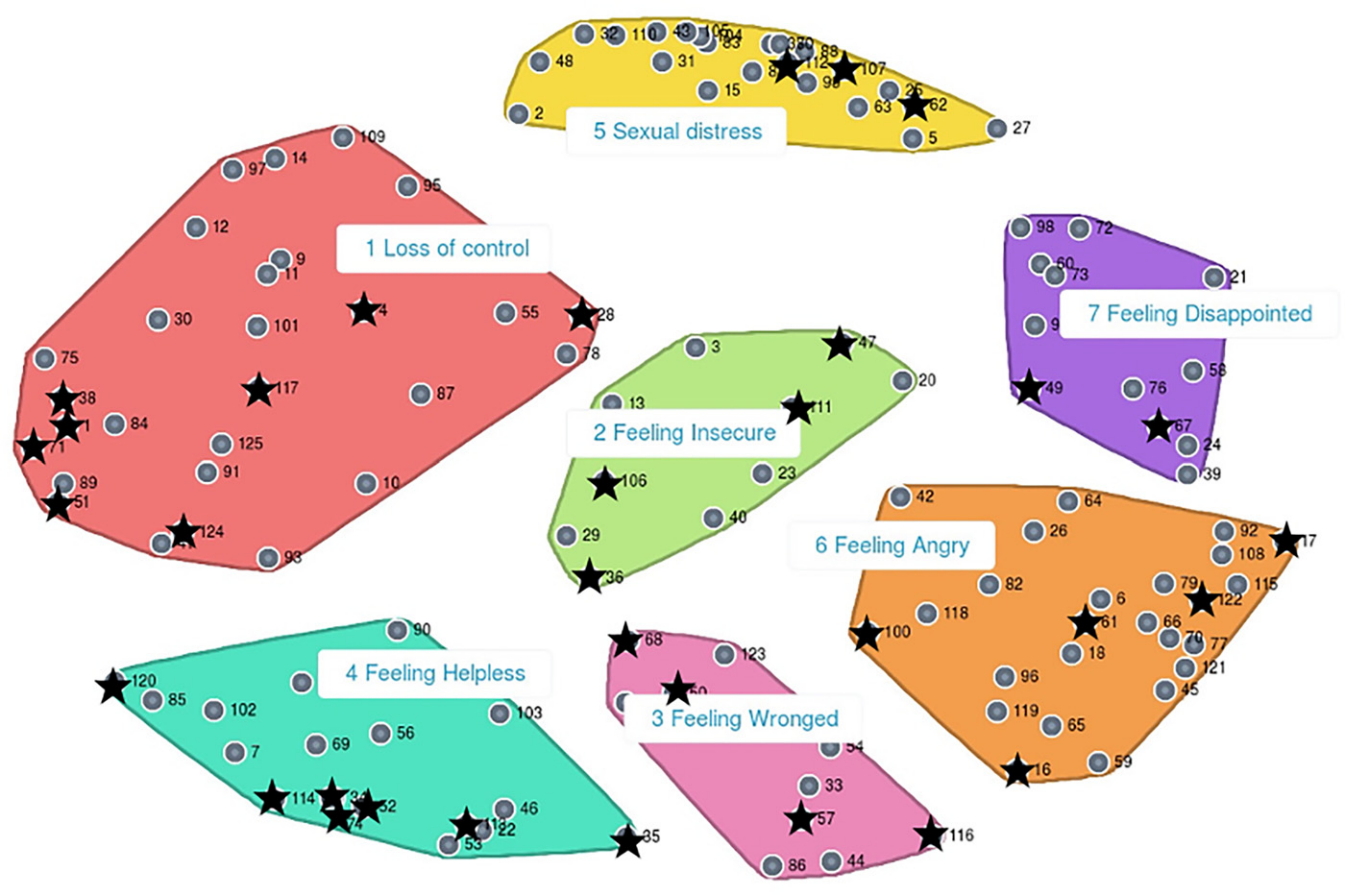
The point-cluster map as conceptual model. Note: The stars indicate the position of the selected statements that represent women's sexual and psychological burden.

During the MDS analysis of the similarity matrix, 10 iterations were needed before it converged and produced a final rather high stress value of 0.36 in comparison to the range found in meta-analyses that examined the results of other concept mapping studies.^49^ Despite its magnitude, the stress value was deemed acceptable. The visualized results of the MDS analysis are shown in Figure 1. All 125 statements are plotted in a 2-dimensional space, depicted in the numbered points in this point map. The distances between points, and between the boundaries of the clusters of points emphasize conceptual meaning when interpreting the concept map.^46^

Figure 1 also shows the HCA results and additionally depicts the thematic areas of the cluster map based on the MDS analyses and results. Each cluster represents a concept that is labeled conform the content and meaning derived from the statements in the cluster. The relative position of clusters provides further information about their relationship with each other.

After reviewing each cluster configuration in the review process described above, the research team agreed on a 7-cluster solution as the most parsimonious description of the content. Label names were chosen based on content, meaning, and the participants’ label suggestions, and translated into English for this article. To illustrate the content of each cluster, Table 1 provides an overview of the cluster labels and definitions.

**Table 1:** Cluster definitions and content

Nr.	Cluster definition	Selected statements per cluster	Selected statements that represent women's sexual and psychological burden
1	Loss of control Being unable to control one's feelings or actions	8 out of 28	I am constantly aware of my physical limitations I am fearful to increase my activity level and consequently having to pay for this I lack control over my body It is tiring to constantly balance my activities I feel hesitant when I cannot do what I want because of physical reasons I have to think ahead to plan my activities well I feel tired because of pain I struggle with whether I should take the risk of provoking my physical complaints or not
2	Feeling Insecure Feeling uncertain or anxious	4 out of 10	I feel it is a sign of weakness to show that I am not well I lack trust in my body I feel stressed about the future I doubt if what I do is right
3	Feeling Wronged Being treated unfairly, or unjustly	4 out of 10	I crawl into my shell when I talk about my physical complaints I feel lonely, home alone in my own small world I feel insecure when women in a similar situation can do things that I cannot do I feel sad to have to say I am not well
4	Feeling Helpless The inability to defend oneself or act without help	7 out of 18	I find it difficult that people underestimate my physical complaints It is challenging to talk about restrictions that I experience I feel irritation over other people's remarks regarding my physical complaints I have to defend myself when I have to explain how I suffer I feel sad that others do not understand I feel sad about other people's judgements I refuse to participate in social activities with friends due to physical complaints
5	Sexual distress A problem occurring during sexual intercourse that results in unsatisfactory sex	3 out of 22	I feel helpless about the influence of my physical complaints on my sex life I feel disappointed about the restrictions in my sex life due to physical complaints I feel sad about the things that I am incapable of doing during sex due to physical complaints
6	Feeling Angry Feeling or showing strong annoyance, displeasure, or hostility	5 out of 25	I feel angry when I lack control over my body I feel angry about the severity of my physical restrictions I feel helpless because I cannot do what I want I feel shocked when my physical complaints suddenly occur I am furious about the relapses of my physical complaints, when I work so hard to recover
7	Feeling Disappointed Feeling sad or displeased because something falls short of someone's expectations	2 out of 12	I am going in circles of fear and pain I feel uneasy when my mobility is limited

Cluster labels and statements were translated from Dutch into English.

In terms of the cluster-map configuration and content, the *Loss of control* cluster contained statements expressing a physical difficulty performing daily and sexual activities, and recovery issues. The cluster occupied the largest space and has a low item density, reflecting a weaker conceptual coherence, based on the judgments of the participants. In contrast, the *Sexual distress* cluster showed a higher item density and overall, the cluster occupied a smaller space indicating a stronger conceptual coherence. The items in this cluster represented physical restrictions performing sexual activities, loss of sexual pleasure, and sexual disappointment. *Feeling Insecure* contained statements expressing a sense of failure, stress and doubts. Typically, a cluster in the center of the map can be indicative of either a set of statements that are ambiguous or difficult to categorize, or a meaningful, coherent concept with a pervasive, connecting relation to all other clusters. The latter appears to be the case in this study. Lack of acceptance, loneliness, sadness, and dilemmas are grouped under the *Feeling Wronged* cluster*,* and feeling misunderstood, combined with feelings of incapability, guilt and trauma under the *Feeling Helpless* cluster*.* Feelings of anger, fury, unfairness, uselessness, and surprise were grouped in the *Feeling Angry* cluster*.* Finally, the *Feeling Disappointed* cluster contained mainly self-image items concerning one's physical appearance, related to relational and sexual activities. The latter 4 clusters appeared to have a blend of areas with high and low item density based on the distribution of points within their respective boundaries.

### Rating

**Figure 2:**
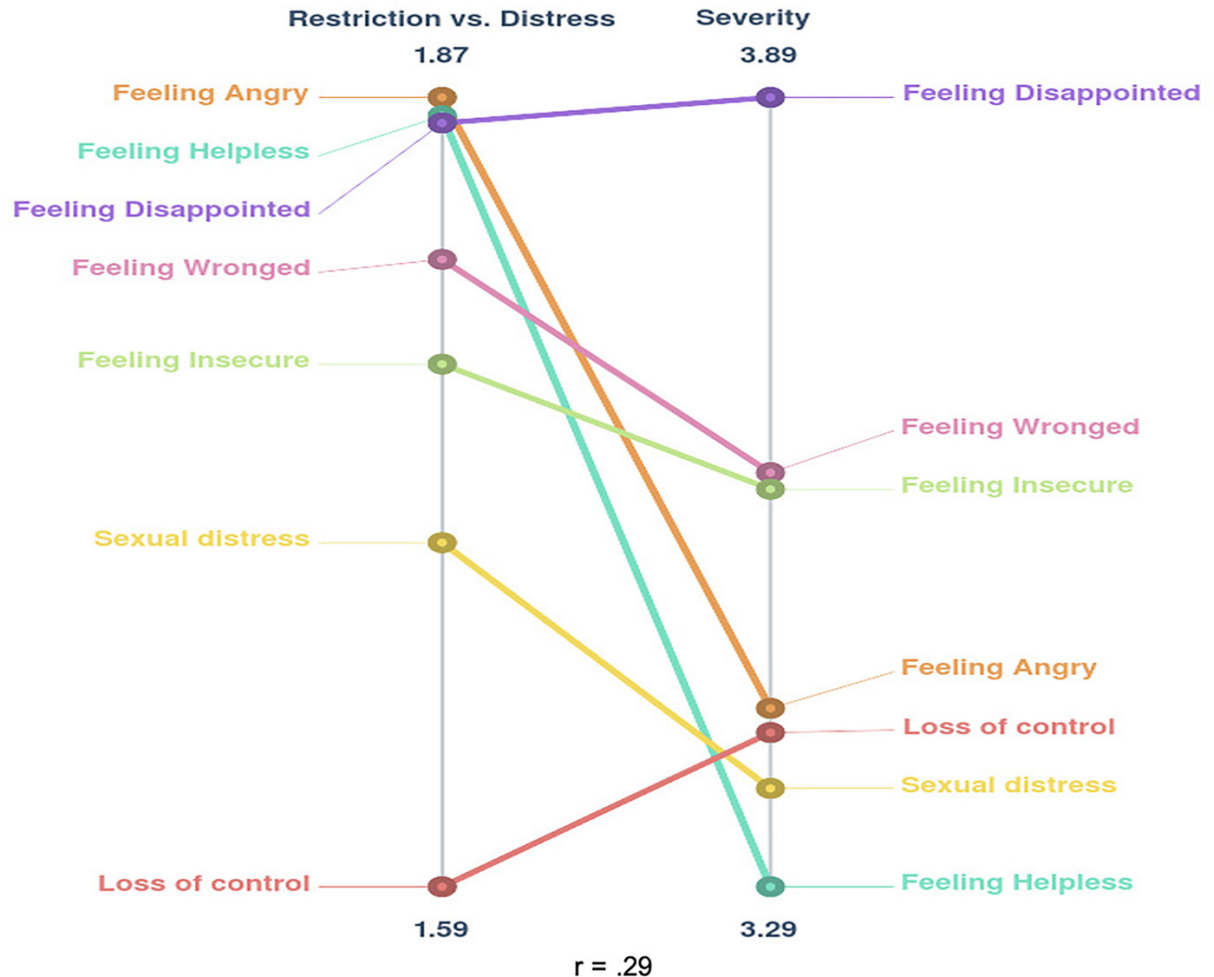
Relative pattern match of mean rating scores.

Across participants, averaged rating scores were calculated for each statement and cluster to examine different value patterns across the conceptual model. The pattern match (Figure 2) revealed the limited graphical and statistical correspondence between the patterns of the nature and severity ratings. Comparison of the average cluster nature and severity ratings showed a substantial variation in the order of the clusters. A correlation coefficient of r = 0.29 confirmed the lack of congruence in the 2 rating patterns.

The participants rated feeling angry, helpless, and disappointed at the top end of the restrictions vs distress scale. In addition, feeling disappointed was also rated as the most severe type of distress. Feeling angry and helpless were valuated as substantially less severe. Consequently, the participants qualified disappointment as an important, overwhelming, and conspicuous distress, whereas feeling angry and helpless appear to be more common or acceptable types of distress in women with pelvic floor complaints.

### Statement Selection

In the severity rating dataset for statement selection, the observation of 13 missing values would have resulted in the exclusion of the scores of 3 experts for the item-total correlation computation. Little's MCAR test^50^ was performed, which proved not significant (*P* = 1.00). This suggested that the values were missing at random. Therefore, missing values were imputed with the series means to complete the dataset.

Calculation and analyses of the item-total correlations of the severity ratings were performed in SPSS-26. Fifteen statements were initially identified using the 0.70 item-total correlation selection criterion. The statements were reviewed relative to the concept map to examine the distribution of items across the emergent conceptual model. Because this initial short list did not represent statements from all 7 clusters, the selection threshold was lowered to 0.60 to prioritize content from all clusters. This resulted in the selection of 33 statements, which represented each cluster with at least 2 statements. An overview of the selected statements is shown in Table 1. These statements represent the most prominent types of distress according to the participants and are indicative of the content of the respective clusters. They are not perfect descriptors of the given label, but the result of exploratory rather than confirmatory research. The selected statements’ origin and distribution were then checked on the conceptual model, and they are indicated on the concept map with black stars in Figure 1.

## DISCUSSION

A conceptual model of women's sexual and psychological burden with pelvic floor complaints was developed, based on interview data in a previous mixed-method study,^39^ and the perspectives of women with pelvic floor complaints and different health care providers. Statements that can be used for an inventory assessing the sexual and psychological burden emerging from physical complaints were statistically derived and conceptually refined.

Six out of 7 types of distress from the conceptual model were also found in the previous text-mining study.^39^ In the present study, 1 type of distress *(Feeling insecure)* that emerged close to the center of the cluster map (Figure 1) did not appear to be an important type of distress in the previous study, although it was entered as a concept in the analyses.^39^ Remarkably, this cluster's central position on the cluster map suggests a broad and pervasive connection with the other 6 types of distress. In both studies, *Sexual Distress* and *Feeling Disappointed* were important types of distress in all interviewed participants.^39^ *Feeling Wronged, Feeling Helpless,* and *Feeling Angry* were important types of distress in women who sought help. *Loss of control* was an important issue in pregnant women.^39^ The fact that the majority of the distress types that emerged in this study were prevalent in women who sought help, might be explained by the participants’ personal experiences with pelvic floor complaints, and the fact that health care providers only meet the women who seek help.

Commonly reported types of distress in previous studies in this field of interest are insecurity or lack of self-confidence, and anger, which correspond with the present study's outcomes. However, the present study results showed that insecurity may have a pervasive character and provide an underlying basis for other types of distress. In the text-mining study, embarrassment was an important type of distress in nulliparous women, as was guilt in pregnant women. Feeling depressed and being self-conscious were important themes in the narratives of women receiving pelvic physical therapy.^39^ Fear, anxiety, frustration, and shame did not qualify as important types of distress in women with pelvic floor complaints, despite the fact that they were included in the 57 concepts in the previous study and the 125 statements in this study. These findings imply that some of the distress types referred to in the literature are not reported by all women with pelvic floor complaints, but that they are more subgroup- or context-related. A striking finding is that feeling disappointed emerged as an important and severe distress in this study, as well as in the text-mining study, whereas it was not reported in the literature.

These observed discrepancies between the findings of other researchers and outcomes of this and the text-mining study indeed raise questions. Firstly, if researchers’ and healthcare professionals’ expectations and interpretation of women's distress with pelvic floor complaints are such that women's distress might be overrated or underestimated, how does this affect the care that is given? Secondly, what is the standard and quality of communication on this topic? Miscommunication may be due to a lack of awareness regarding the sexual and psychological burden of women with their physical complaints, or ignorance about appropriate questions to ask. Therefore, the list of statements assessing the sexual and psychological burden with physical complaints might contribute to improving communication on this topic, and ultimately to care given to women with pelvic floor complaints.

### Limitations

One of the main limitations is the binary rating option for the statements as restriction or distress. The resulting lack of similarity between both rating values in the pattern match could be explained as an artifact of measurement or scaling and renders a measurable difference uncertain. In hindsight, the binary choice option in rating the statements as restriction or distress is merely an indicator of distinction, instead of importance or value. Following the rationale that 1.5 is the cutoff value for restriction or distress, these scores should be interpreted in relation to this figure. Consequently, lower mean scores depict a tendency to classify the cluster as a restriction, and higher mean scores depict a tendency towards classification of the cluster as a type of distress. Effectively, none of the clusters was classified as a restriction. In comparison, the use of a 5-point Likert-type scale for rating the statements’ severity is more indicative of value, which may explain why the average rating values are dissimilar and incompatible. Despite the initial logic to question the statements’ nature binary, the use of a 5-point Likert-type scale in this rating task could have improved comparative compatibility.

Another limitation is the high stress value. A high stress value indicates a lower fit in the MDS output that could be based on variability in interpretation and valuation of the statements.^49^ Sorting ambiguity or differences in participants’ characteristics or background can influence the height of the stress value. Therefore, the diversity of the group of participants may account for this limitation. Participants who personally experienced pelvic floor complaints may have recognized their own distress during the sorting and rating tasks, and thus underlined women's psychological burden. It can, however, be argued that the health care professionals may not have provided an accurate reflection of women's psychological burden. Not only because of (possible) lack of personal experience with pelvic floor complaints or gender but also because of the potential risk of misinterpretation and valuation of the statements. This may, in effect, have influenced the content of the inventory as well. However, input from this varied group of participants was deliberately sought, and is indicative of the level of acknowledgement and interpretation of women's distress.

### Future Research

This study could be replicated including a larger number of women with pelvic floor complaints and health care providers. Data from both groups could then be compared, which may provide further insight into the congruence of the interpretation of the given statements, and into between-group discrepancies. As discussed earlier, to improve the interpretability of the outcomes, the binary restriction or distress rating option should be adapted.

## CONCLUSION

This study's outcomes add to more in-depth insight into women's sexual and psychological burden with pelvic floor complaints and provide a more comprehensive overview of this burden in comparison to previous research on this topic. 7 thematic distresses were identified: loss of control, feeling insecure, feeling wronged, feeling angry, feeling helpless, feeling disappointed, and sexual distress. Feeling insecure appears to have a central and pervasive relation with the others. The overlap in outcomes with previous mixed methods studies indicates a promising perspective the sexual and psychological burden with pelvic floor complaints that is shared by the women who suffer from these complaints and the professionals who provide them care. The list of statements may help future research aimed at developing an instrument to assess the type and severity of distress experienced by women with pelvic floor complaints.

## ACKNOWLEDGMENTS

The authors would like to thank the participants for their contribution to this research.

## STATEMENT OF AUTHORSHIP

Alma Brand: Conceptualization, Methodology, Software, Validation, Formal Analysis, Investigation, Resources, Data Curation, Writing - Original Draft, Visualization, Project Administration; Scott Rosas: Software, Validation, Formal Analysis, Investigation, Resources, Writing - Review & Editing, Visualization, Supervision; Wim Waterink: Conceptualization, Writing - Review & Editing, Supervision, Project Administration; Slavi Stoyanov: Conceptualization, Methodology, Software, Formal Analysis, Writing - Review & Editing, Supervision; Jacques van Lankveld: Conceptualization, Writing - Review & Editing, Supervision, Project Administration.
